# Association of the conicity index with diabetes and hypertension in Brazilian women

**DOI:** 10.1590/2359-3997000000187

**Published:** 2016-08-23

**Authors:** Mirelli Dantas Andrade, Maria Camila Pruper de Freitas, Alyne Mayumi Sakumoto, Caroline Pappiani, Samantha Caesar de Andrade, Viviane Laudelino Vieira, Nágila Raquel Teixeira Damasceno

**Affiliations:** 1 Departamento de Nutrição Faculdade de Saúde Pública Universidade de São Paulo São Paulo SP Brasil Departamento de Nutrição, Faculdade de Saúde Pública, Universidade de São Paulo (FSP/USP), São Paulo, SP, Brasil; 2 Centro de Saúde Escola Geraldo de Paula Souza Faculdade de Saúde Pública Universidade de São Paulo São Paulo SP Brasil Centro de Referência para a Prevenção e Controle de Doenças Associadas à Nutrição (CRNutri), Centro de Saúde Escola Geraldo de Paula Souza (CSEGPS), Faculdade de Saúde Pública, Universidade de São Paulo (FSP/USP), São Paulo, SP, Brasil

**Keywords:** Conicity index, diabetes, hypertension, hyperglycemia

## Abstract

**Objective:**

The goal of this study was evaluate the conicity index (C index) in women and its association with hypertension (SAH) and diabetes mellitus (DM).

**Subjects and methods:**

This was a cross-sectional study, with 573 women between 20 and 59 years of age. After analysis of clinical and demographic characteristics, anthropometric variables were measured and used to calculate the C index. Plasma glucose and lipid profile were evaluated by standard methods. The analysis of the results was based on logistic regression and the odds ratio (OR) was calculated, which was used to assess the association of the variable outcome with the variable exposure using two logistic regression models that tested the possible influence of the C index in the chance of developing SAH or DM. A confidence interval of 95% was used.

**Results:**

In the crude and adjusted models, the OR confirmed the association of the C index with DM and SAH. Compared with women that showed C index p < 75, the risk of women with C index (p ≥ 75) developing DM and SAH was 1.72 and 1.75, respectively. Results demonstrated that the negative impact of age on these associations significantly raised the odds of women having DM and SAH. The high C index was also linked to low HDL-C.

**Conclusion:**

The C index is an important tool in estimating the risk of diabetes and hypertension in women. Besides, high C indexes are negatively associated with HDL-C, an important lipid marker related to cardiovascular risk.

## INTRODUCTION

Cardiovascular disease (CVD) is the main cause of early morbidity and mortality all over the world, significantly affecting the resources of public policy programs. Although CVDs are multifactorial, it is known that systemic arterial hypertension (SAH) and diabetes mellitus (DM) are, respectively, the first (13%) and the third (6%) cardiovascular risk factors in the worldwide population (1). Besides these factors, overweight and obesity have an important negative role, as they are, together, responsible for 5% of the CVDs. Following the global trend, in 2012, CVDs were responsible for 33% of all deaths and 74% of the deaths caused by chronic non-transmissible diseases in Brazil (1).

It is know that obesity has a direct impact on the development of CVD, and it is an aggravating circumstance for risk factors such as dyslipidemias (DLP), SAH, and DM. According to the World Health Organization (WHO), increased obesity is also one of the main health concerns worldwide. It is estimated that, in all regions of the world, obesity has doubled from 1980 to 2008 and, in 2012, it was estimated that obesity affected half billion people (12% of the population) (1).

Considering the role of obesity in CVD, Valdez proposed the Conicity Index (C Index), which was developed as an indicator of obesity and body fat distribution (2). This index considers that central obesity, more than generalized obesity, is associated with the development of CVD (3,4).

The C index is based on the hypothesis that people that accumulate fat around the abdomen have a shape similar to a double cone (that is, two cones sharing the same base, one placed over the other), whereas those people that have less fat in the central region have the shape of a cylinder. Therefore, C index estimation uses variables such as weight, height, and abdominal circumference (AC). The index is calculated using the formula below (4,5).



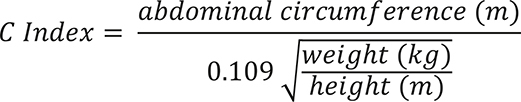



The numerator is CA in meters; 0.109 is the constant that results from the squared-root of the ratio between 4π (from the deduction of the perimeter of a cylinder) and mean human density (1.050 kg/m^3^). The denominator is the cylinder produced by the weight and height of the individual. In theory, the C index ranges from 1.0 (a perfect cylinder) to 1.73 (a perfect double cone), and values increase according to the accumulation of fat in the central region of the body. That is, the closer to 1.73, the greater the accumulation of abdominal fat (4,5).

Considering the negative impact of excess body weight in the development of CVD, several studies have been conducted to identify the possible associations between the C index and cardiovascular risk factors (3,6,7). In Brazil, the study by Pitanga and Lessa demonstrated that the C index was the indicator of central obesity that best discriminated high cardiovascular risk in males (8). Observational studies with adult individuals of both sexes showed that the C index was directly associated with total cholesterol (CT), triglycerides (TG), visceral fat, and inversely associated with high-density lipoprotein cholesterol (HDL-C) (9,10). Therefore, the C index is a potential clinical tool to be applied to the evaluation of cardiovascular risk in a population. Besides the association of the C index with a more atherogenic lipid profile, this index is also associated with changes in blood glucose and blood pressure (11).

Although the C index is a clinical tool for the estimation of cardiovascular risk that is useful, low-cost, and easy to be applied, few Brazilian studies have used this indicator, mainly in the estimation of cardiovascular risk in women. Therefore, the objective of this study was to evaluate the C index in female outpatients and the possible association between the index and SAH and DM.

## SUBJECTS AND METHODS

### Experimental design and sampling procedure

The study was a cross-sectional one based on a subsample of the study “Effectiveness of nutritional intervention focusing on prevention and control of diseases and non-transmissible disorders in a basic health unit”, which was previously approved by the Research Ethics Committee of the *Faculdade de Saúde Pública* at *Universidade de São Paulo* (FSP-USP) (Of. COEP/168/11), and according to ethical requirements of *Resolução* CNS nº 466/12 and its complementary resolutions. The study included adult women between 20 to 59 years of age that were seen at the *Centro de Referência para a Prevenção e Controle das Doenças Associadas à Nutrição* (CRNutri), which is part of the *Centro de Saúde Escola Geraldo de Paula Souza* (CSEGPS), from FSP-USP. Data were collected from August 2000 to July 2014, and all participants signed an Informed Consent Form.

### Characteristics of the sample

Sociodemographic and clinical information were obtained for the occurrence of SAH, DM, and DLP, as well as the use of drugs for SAH, DM, and DLP. The use of drugs was self-reported or determined by patient records.

Patients were excluded from the study if they showed acute inflammatory diseases, uncontrolled diseases, psychiatric diseases, history of cardiovascular events, if they were pregnant or breastfeeding, or if they presented any disease that made it impossible to collect anthropometric data necessary for the evaluation of the nutritional status.

### Anthropometric assessment

The techniques proposed by the *Sistema de Vigilância Alimentar e Nutricional – Ministério da Saúde* (SISVAN – MS) were used in the assessment of height, weight, and AC (12). Weight was assessed on a calibrated digital scale (Toledo do Brasil^®^, model 2096 PP/02, maximum weight of 200 kg and readability of 100 g); height was determined in meters using a stadiometer of maximum height 2.1 m and readability of 1 mm fixed to a smooth wall without a baseboard (Sanny Standard). Body mass index (BMI) was calculated and patients were classified according to the WHO standards (13). AC was measured with the patient in standing positive, with parallel feet and a relaxed abdomen. The navel was used as a reference, and the measure tape was placed parallel to the ground (12).

### Biochemical assessment

Blood samples were collected by venipuncture after 12-hour fast. Biochemical assessment of blood glucose, TC, LDL-C, HDL-C, and TG was carried out by means of commercial kits and standard methods.

### Statistical analyses

Statistical analyses were carried out in the Statistical Package for the Social Sciences (SPSS) version 20.0. For qualitative variables, χ^2^ was used and results are presented as absolute values followed by their respective percentage. For quantitative data, as well as for the determination of the tests to be used, the type of variable distribution was determined by Kolmogorov-Smirnov test (p > 0.05). Variables with normal distribution are presented as mean and standard deviation, and means were compared by Student t test. For the other variables, values are presented as medians and interquartile ranges, and Wilcoxon non-parametric test was used. Significance level was set at p < 0.05.

SAH and DM were considered as the dependent variables in the regression tests. SAH was a dichotomous variable: all participants that reported the use anti-hypertensive drugs were classified in the category “yes”, and those that did not report using these drugs were classified in the category “no”. As for DM, it was also a dichotomous variable based on the reference values published by the guidelines of the *Sociedade Brasileira de Diabetes* (SBC, 2014) (14). Participants that showed fasting blood glucose ≥ 100 mg/dL were classified in the category “yes”, and those that presented normal values were classified as reference “no”. C index was considered an independent variable. Based for the results of the C index, the sample was stratified in percentiles, and two groups were formed: group C index p < 75 (first, second, and third quartile) and group C index p ≥ 75 (last quartile). Therefore, a dichotomous variable was generated in a way that the group C index p ≥ 75 was classified in the category “yes” and the group C index p < 75 was classified as reference “no”. The odds ratio (OR) was estimated to analyze the association between the outcome variable and the exposure variable, using two logistic regression models in which the possible influence of C index on the occurrence of SAH and DM was analyzed. In the multiple model, age was a co-variable. A 95% confidence interval was used.

## RESULTS

The study analyzed 573 women with median age 49.2 (41.4-54.6) years. The demographic, anthropometric and clinical characteristics are presented in Table 1. Median weight and BMI of the group C index p ≥ 75 were higher than C index p < 75; the same occurred with the AC mean. In relation to the presence of chronic non-transmissible diseases 38.4% showed SAH, and 20% presented DLP. The frequency of DM in the group C index p ≥ 75 was 22.2%.

**Table 1:** Demographic, anthropometric, and clinical characteristics of the participants of the study, São Paulo, 2014

Variables	Total (n = 573)	C index p < 75 (n = 429)	C index p ≥ 75 (n = 144)	p
Age (years)	49.2 (41.4-54.6)	48.5 (40.4-54.3)	50.9 (44.4-55.3)	0.015
Weight (kg)	72.2 (63.5-83.5)	70.1 (60.8-80.6)	78.6 (70.7-92.6)	< 0.001
BMI (kg/m^2^)	29.3 (25.7-33.2)	28.2 (25.0-32.0)	32.0 (29.3-36.6)	< 0.001
AC (cm)	98.8 (13.6)	94.5 (11.2)	111.6 (12.0)	< 0.001
Diseases*				
SAH (n, %)	220 (38.4)	148 (34.5)	72 (50.0)	0.001
DM (n, %)	74 (12.9)	42 (9.8)	32 (22.2)	< 0.001
DLP (n, %)	115 (20)	9 (2)	106 (74)	< 0.001

Results presented as means and standard deviations (parametric variables); medians and interquartile range (non-parametric variables); and absolute values (n) and frequencies (%) (qualitative variables). BMI: body mass index; AC: abdominal circumference; SAH: systemic arterial hypertension; DM: diabetes mellitus; DLP: dyslipidemias.

* Diseases classified based on reported occurrence and use of medical treatment.

Difference between the groups determined by Student t test for parametric variables, Mann Whitney test for non-parametric variables and χ^2^ test for categorical variables. Statistical significance set at p < 0.05.

**Graph 1:**
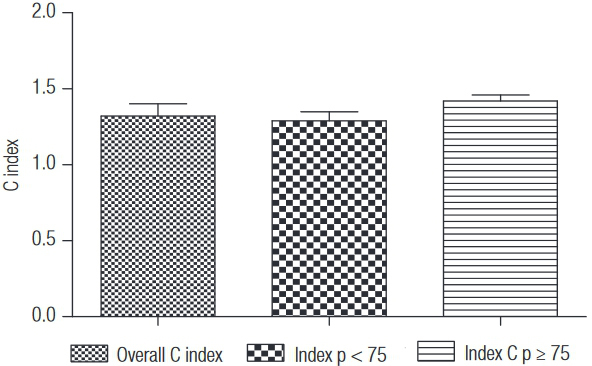
Overall C index, C index p < 75, and p ≥ 75, São Paulo, 2014.

Mean C index in the overall sample was 1.32 (0.08). After the stratification, women in group C index p < 75 showed mean equal to 1.29 (0.06), and in group C index ≥ 75 mean was 1.42 (0.04) (Graph 1).

The biochemical profile of the women in the present study is shown in Table 2. It may be observed that fasting blood glucose in the group C index p ≥ 75 was greater than 99 (89-114) mg/dL, compared with C index p < 75. However, HDL-C was lower in the group C index ≥ 75.

**Table 2:** Biochemical characteristics of the subjects, São Paulo, 2014

Variables	Total (n = 573)	C Index p < 75 (n = 429)	C Index p ≥ 75 (n = 144)	p*
Blood glucose (mg/dL)	95 (86-107)	94 (86-104)	99 (89-114)	**< 0.001**
TC (mg/dL)	211 (44)	211 (81-393)	209 (106-317)	0.545
LDL-C (mg/dL)	135 (39)	135 (41)	134 (36)	0.714
HDL-C (mg/dL)	46 (40-55)	47 (40-57)	44 (38-51)	**0.004**
TG (mg/dL)	121 (86-173)	120 (83-172)	125 (96-178)	0.117

Results presented as means and standard deviations (parametric variables), and medians and interquartile range (non-parametric variables). TC: total cholesterol; LDL-C: low-density lipoprotein cholesterol; HDL-C: high-density lipoprotein cholesterol; TG: triglycerides. * Difference between the groups determined by Student t test for parametric variables, and Mann Whitney test for non-parametric variables. Statistical significance set at p < 0.05.

**Figure 1:**
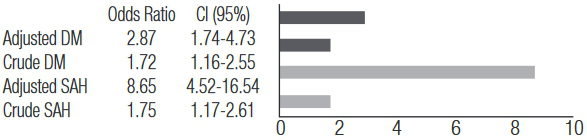
Crude odds ratio and odds ratio adjusted by age with respective confidence intervals (CI 95%), according to SAH and DM.

Crude and adjusted models showed that OR (Figure 1) confirmed the association between the C index with DM and SAH. The chance of showing DM and SAH in participants of group C index p ≥ 75 was, respectively, 1.72 and 1.75 times greater than that of patients in group C index p < 75. The model adjusted by age showed that chances increased to 2.87 and 8.65, respectively, demonstrating that the influence of the C index in DM and SAH increased with age.

## DISCUSSION

Results presented here demonstrated that the C index was associated with low plasma concentrations of HDL-C. It was also observed that women with high C indexes showed 72% and 75% more chance of having DM and HAS, respectively. The negative impact of age on these associations significantly raised the chance of these women having DM and HAS.

In the study by Ghosh and cols. (15), which compared the association of obesity indicators and eating habits with metabolic risk factors for coronary heart disease, researchers found results similar to ours, where the C index was also associated with high blood glucose. The authors also found associations with high levels of TG and TC, proposing that the C index was the most consistent indicator in the explanation of metabolic variables of coronary heart disease.

In 2002, Pitanga and Lessa (16) found a prevalence ratio of 1.34 (1.02-1.75) between the C index and systolic arterial pressure (SAP) in men over 50 years of age, and of 2.20 (1.40-3.46) in women less than 50 years old, after adjusting to blood glucose. Similarly, in our study, the C index was associated with DM and SAH. Together, these results suggest that the C index may be used as a predictor of CVD (17).

The distribution of abdominal fat is associated with metabolic changes and increases the risk of CVD and type 2 DM, and it is important to identify high-risk individuals for CVD prevention. Anthropometric assessment is considered an efficient, non-invasive and low-cost method; however, not all anthropometric parameters are efficient in the evaluation of abdominal obesity (17).

Previous studies have demonstrated that other anthropometric parameters are also associated with a more atherogenic lipid profile. The BMI is widely used in population studies (7), although its ability to estimate body fat distribution and organ fat is controversial and limited (5). On the other hand, measurements such as waist circumference (WC) and waist-to-hip ratio (WHR) have been analyzed to determine their ability to estimate central fat, although WHR sensitivity has been questioned. The C index may be a useful tool in the evaluation of abdominal obesity (9).

Zhou and cols. evaluated, in more than 29000 individuals, the association between BMI, WC, WHR, and the C index with arterial blood pressure. In this study, the authors observed that WC and WHR were more strongly associated with blood pressure and SAH, when compared with BMI. This association was more intense in men than in women. On the other hand, our study showed that women with C index ≥ p75 presented greater chances of having SAH (18).

Afzar carried out a study in order to analyze the specific relationships between anthropometric parameters such as BMI, WC, WHR and C index with some types of SAH, such as sustained normotension (SNT), white-coat hypertension (WCHT), masked hypertension (MHT), and sustained hypertension (SHT) with patients recently diagnosed with type 2 DM. The author demonstrated that only WC and WHR presented significant differences in SNT, WCHT, MHT, and SHT in these patients. According to the regression analysis in the study, only BMI was independently related with MHT. The C index did not show any differences and the author questioned if this index, which estimates abdominal fat and cardiovascular risk, was not able to identify SAH (19).

In another study that aimed at comparing the C index and BMI as a discriminator of hyperglycemia in 1,325 adults in the city Salvador-BA, the C index showed greater discriminatory power than BMI, with areas under the ROC curve equal to 0.71 (0.66-0.77) and 0.62 (0.57-0.67), respectively (20). Similarly, our study demonstrated that the chances of the participants of group C index p ≥ 75 presenting DM was 1.72 times greater than the chances of participants of group C index p < 75.

A study by Almeida and cols. assessed the association between abdominal obesity and cardiovascular risk in women, and showed that the C index was the indicator with the best performance in the discrimination of coronary heart disease risk (21).

Given this scenario, Pitanga e Lessa carried out a study that aimed at proposing cutoff points for the C index. The authors concluded that the best cutoff points were 1.25 and 1.18 for males and females, respectively, based on the results of the analysis of the ROC curves for sensitivity and specificity of the best cutoff points for the C index as a discriminator of high coronary heart disease risk (HCR) (3). The authors concluded that the C index may be used to discriminate high cardiovascular risk, although sensitivity and specificity were not very high. In men, both sensitivity and specificity were about 75%, and the C index may incorrectly identify 25% of the patients with HCR, and 25% of those with “normal” cardiovascular risk. On the other hand, in women, the most adequate cutoff point to discriminate HCR shows sensitivity of about 73% and specificity of about 61%. Therefore, the possibility of incorrect classifications is greater in those with low cardiovascular risks, leading to greater number of false-positive results. Taking into account the limitations cited here, we opted, in our study, to stratify the sample starting in the 75^th^ percentile, as only 4% of the women presented C index < 1.18, making it impossible to use the currently proposed cutoff point.

Vidigal evaluated the ability of the anthropometric indicators to discriminate cardiovascular risk with high levels of C-reactive protein (CRP) and fibrinogen in 130 adult men. The results of the study pointed out that the C index showed the greater ability to detect high levels of CRP and fibrinogen, which are related with increased cardiovascular risk, compared with the other anthropometric indicators, such as BMI, WC, and WHR (17).

In a study carried out with men and women, which analyzed the precision of four anthropometric indicators of obesity (BMI, WC, WHR, and C index) in the identification of SAH, it was observed that, in both sexes, all indicators had a satisfactory ability to detect the presence of SAH. The association between obesity and SAH was greater in women than in men, and a possible explanation for these results is the use of oral contraceptives by women, which increases the risk of SAH (22).

In a study that aimed to assess the relationship between the C index and SAH in 72 middle-aged and elderly women using the 1.18 cutoff point, the C index was positively correlated with SAP and diastolic blood pressure (DAP). Logistic regression identified two significant odds ratio for the increased C index, demonstrating that the cutoff point was efficient in identifying its relationship with SAH in the population studied (23).

In menopause, changes in the distribution of body fat lead to increased cardiovascular risk and metabolic diseases. Organ fat is highly associated with changes in lipid and carbohydrate metabolism (24). Our results show that the greater the C index, the greater the chance of diabetes in the women.

In a study carried out with 169 post-menopausal women, WC was significantly correlated with SAP and DAP. On the other hand, BMI was significantly correlated only with SAP. The C index showed significant correlation with SAP. The results of our study showed that the chances of a woman being hypertensive increase with greater C indices. Besides, our study analyzed a sample that was larger than that of the study cited (24), reinforcing the validity of the results presented here.

Other authors observed that WC is an independent predictor of high metabolic risk and insulin resistance in Chinese, post-menopausal women (25). Hwu and cols. observed a stronger association between anthropometric indicators of obesity and cardiovascular risk in younger women compared with older ones. According to the authors, the results may be explained by the typical hormonal changes of menopause, more prevalent at this age. In menopause, women are more vulnerable to metabolic disorders, such as DLP, and they may increase the risk of coronary heart disease (26).

### Limitations

One limitation of our study is related with the nature of cross-sectional studies, which are not able to determine temporality. We suggest that future, prospective studies are carried out in order to establish a causal relationship.

### Advantages of the study

The C index showed to be an important indicator of fat distribution, demonstrating changes in it, and enabling comparisons between individuals that show different body weights and heights. Given the results obtained here, it is recommended that the C index is included in the overall risk evaluation for DM and SAH in women. This index showed to be a useful clinical tool in the estimation of cardiovascular risk, being of low-cost and easy to be applied. Until now, few Brazilian studies have used this indicator in the estimation of cardiovascular risk, mainly in women, whose risk is known to increase with age.

## CONCLUSIONS

The results of the present study showed that the chances of women having DM and SAH increase with the C index (p ≥ 75). Besides, the negative impact of age on these associations significantly increases the chances of women having DM and SAH. The C index was also associated with low HDL-C plasma concentrations, and may be an alternative to the anthropometric indicators of obesity associated with risk of coronary heart disease. The results suggest that the C index may be used as an indicator of central obesity and the morbidities associated with it.
